# Functionalizing Nisin with a Sugar Moiety Improves Its Solubility and Results in an Altered Antibacterial Spectrum and Mode of Action

**DOI:** 10.1021/acssynbio.5c00353

**Published:** 2025-08-12

**Authors:** Longcheng Guo, Oscar P. Kuipers, Jaap Broos

**Affiliations:** Department of Molecular Genetics, Groningen Biomolecular Sciences and Biotechnology Institute, 3647 University of Groningen, Groningen 9747 AG, The Netherlands

**Keywords:** RiPPs, nisin, noncanonical amino acid, click chemistry, glycosylation, solubility

## Abstract

Glycosylation, a widespread post-translational modification, is present in all kingdoms of life. Despite the extensive structural diversity found in ribosomally synthesized and post-translationally modified peptides (RiPPs), only a few glycosylated bacteriocins, known as glycocins, have been identified. Notably, glycocins such as glycocin F, ASM1, and enterocin F4-9, exhibit antimicrobial properties and distinct glycoactivity, indicating that glycosylation is crucial for their bioactivity. The development of practical, and widely applicable systems for glycosylation of RiPPs is therefore highly desirable. In this study, we introduce an expression system that utilizes *Lactococcus lactis* as a host for the efficient incorporation of the noncanonical amino acid homopropargylglycine (Hpg) into the well-studied RiPP nisin, and some structurally related variants. Hpg, which has an alkyne functional group, allows for further chemical modifications with azido-sugar containing substrates through click chemistry. We reveal that glycosylated nisin at position 17 shows strong activity against *Enterococcus faecium* strains, but its activity against other pathogens such as *Staphylococcus aureus*, *Enterococcus faecalis*, and *Bacillus cereus* is reduced. Moreover, mode of action studies show that the addition of sugar diminishes its typical pore-forming ability of nisin against *E. faecium* while preserving its lipid II binding ability. Interestingly, the addition of a hydrophilic sugar significantly enhances its water solubility around 4-fold at neutral pH, indicating potential for improved drug applications. These findings highlight the potential of this methodology for glycosylation of RiPPs, leading to the creation of new antimicrobial products with varied characteristics. This also broadens the toolkit for enhancing and discovering peptide-based drugs.

## Graphical abstract

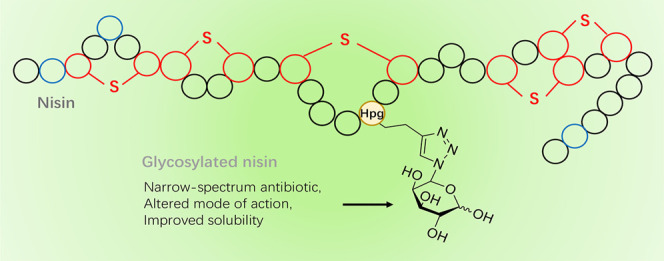

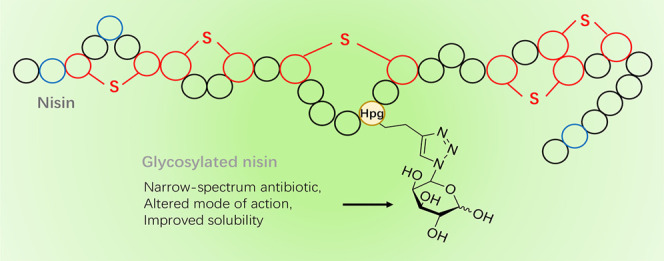

## 1 Introduction

With traditional antibiotics proving inadequate against numerous drug-resistant pathogens, there has been a surge in efforts to discover novel antimicrobial agents and improve existing antimicrobial peptides.^1^ Ribosomally synthesized and post-translationally modified peptides (RiPPs) represent a rapidly expanding class of natural products characterized by diverse structures and biological activities.^2^ They are emerging as promising candidates for combating drug-resistant pathogens due to their unique modes of action.^3^

Nisin,^4^ the first RiPP to be characterized, is primarily produced by*Lactococcus lactis* strains (Figure 1). It is a natural food preservative and has demonstrated antimicrobial activity against a range of food-borne pathogens and spoilage organisms.^5^ Resistance to nisin is slow to develop due to its dual mechanisms of action: it binds to lipid II, interfering with cell wall biosynthesis, and forms pores in cell membranes, causing cellular components to leak out.^6^ The therapeutic use of nisin is being explored in human and veterinary medicine.^7^ For the successful clinical development of nisin, certain challenges such as its limited stability and solubility at physiological pH need to be addressed.^4^ Its ribosomal biosynthesis makes it suitable for genetic code engineering.^8^ The biosynthesis of nisin initiates with the ribosomal production of a precursor, encoded by the *nisA* gene. This precursor is composed of a leader segment and a core peptide segment. The leader segment guides the precursor to NisB for the dehydration of serines or threonines, resulting in dehydroalanines (Dha) or dehydrobutyrines (Dhb). Dha and Dhb can be linked to cysteine catalyzed by NisC to form (methyl)­lanthionine rings.^9^ These extensive postmodifications furnish nisin with rigid ring structures, a key feature for its stability and bioactivity.

**Figure 1:**
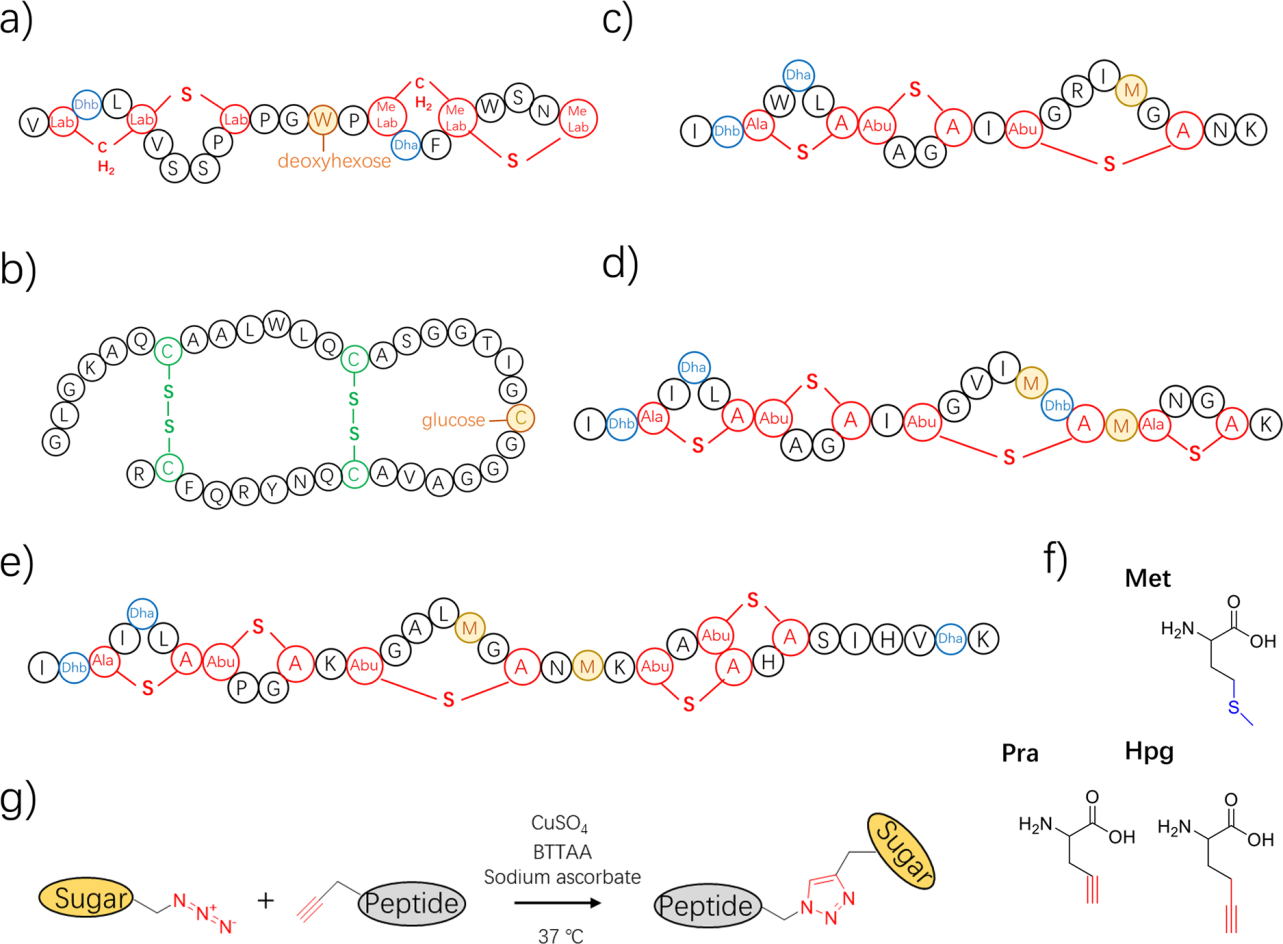
Structures of some glycocins, nisin, nisin variants and the click chemistry reaction. (a) NAI-112, a lanthipeptide containing labionine (Lab) and a glycosylated tryptophan residue, produced by *Actinoplanes* sp. strain with potent bioactivity against nociceptive pain. (b) Sublancin 168, produced by *Bacillus subtilis* 168, featuring a β-*S*-linked glucose moiety attached to Cys22. (c) Cesin, a natural variant of nisin produced by *Clostridium estertheticum*, known for its potent antimicrobial activity against major pathogens. (d) Rombocin, a natural nisin variant produced by *Romboutsia sedimentorum*, displaying selective antimicrobial activity against *Listeria monocytogenes*. (e) Nisin A, one of the most extensively studied lantibiotics, mainly produced by various *Lactococcus lactis* strains. (f) Structures of Met and its analogs Hpg and Pra. (g) Reaction of Hpg-labeled peptide with an azido-sugar moiety using copper (Cu^+^)-catalyzed azido-alkyne click chemistry.

Glycosylation is a common post-translational modification of proteins and is found across all kingdoms of life.^10^ While over half of all proteins are predicted to undergo glycosylation, only a few glycosylated RiPP compounds have been identified within the structurally varied RiPP superfamily.^11^ These compounds typically feature glycosylation on Cys, Ser, Thr or Trp residues, resulting in *S*-, *O*-, or *N*-glycosides. Examples include glycocin F^12^ from *Lactobacillus plantarum*, sublancin 168 ^13^ from *Bacillus subtilis* 168, pallidocin^14^ from *Aeribacillus pallidus* and NAI-112 ^15^ from *Actinoplanes* sp. NAI-112 (Figure 1a) carries a 6-deoxyhexose moiety *N*-linked to a tryptophan residue. Sublancin 168 (Figure 1b) and pallidocin each have a single glycosylated Cys residue, while glycocin F contains both glycosylated Ser and Cys residues. Additionally, there are instances of disaccharide containing glycocins, such as listeriocytocin^16^ and enterocin 96,^17^ both modified at Ser. The lassopeptide pseudomycoidin also exhibits mono- or diglycosylation via a C-terminal phosphorylated serine.^18^ Nonribosomal peptides like vancomycin^19^ from *Amycolatopsis orientalis* and teicoplanin^20^ from *Actinoplanes teichomyceticus*, which feature O-glycosylations, are clinically utilized to combat infections caused by Gram-positive bacteria.^21^

Glycosylation of therapeutic peptides and proteins is a powerful means for many drugs to favorably influence key properties like proteolytic- and thermo-stability, solubility, propensity of aggregation, immunogenicity, as well as offering a tool for improved drug targeting.^22,23^ The employment of a chemoselective reaction, like the copper (Cu^+^)-catalyzed azido-alkyne click chemistry reaction (Figure 1g) allows site specific labeling of a protein, containing an alkyne group harboring noncanonical amino acid (ncAA) with an azido sugar under mild reaction conditions.^24^ Azido sugars are relatively easy to prepare and many are commercially available. The noncanonical amino acids (ncAAs) propargylglycine (Pra) and homopropargylglycine (Hpg) (Figure 1f) are Met analogs containing an alkyne functional group and can be incorporated in a target protein using a Met auxotrophic expression host.

To date, engineering the therapeutic potential of a RiPPs via glycosylation has not been reported. Hpg has been incorporated in few RiPPs, namely nisin, lichenicidin, and capistruin but yields were low or not reported.^25–27^ In a proof of principle experiment, Hpg containing lichenicidin was coupled with azido glucose; characterization of the product was limited to a mass spectrometry spectrum and no bioactivity of the adduct was reported.^26^ In this work, nisin and some structurally related variants (Figure 1c–e) are labeled with Hpg and after isolation functionalized with an azido-sugar substrate using click chemistry. The resulting nisin variants with sugars attached were purified and characterized, and their antibacterial spectrum, modes of action, and solubility at neutral pH are reported herein.

## 2 Results and Discussion

### 2.1 A Cross Expression System for Incorporating the Met Analog Hpg into Nisin

Nisin, being a ribosomally synthesized and posttranslationally modified peptide, requires the expression and modification of nisin through the *nisABTC* gene cluster. In the conventional nisin production system, both the *nisA* and *nisBTC* genes are regulated by the P_*nisA*_ promoter on separate plasmids, pNZnisA and pIL3EryBTC.^28–30^ Since Met is crucial for the translation of post-translational modification (PTM) enzymes and transporters, a cross-expression system has been devised to enable the expression of prenisin and PTM enzymes at different times, as demonstrated in previous studies.^25,31^ *L. lactis* strain NZ9000, auxotrophic in Met, was transformed with a plasmid containing the *nisBTC* genes behind the P_*czcD*_ promoter and another plasmid containing the prenisin derivatives expression controlled by the P_*nisA*_ promoter. First, the expression of *nisBTC* is induced by supplementing Met, followed by switching to a new medium devoid of Met but containing a Met analog for the expression of prenisin derivatives (Figure S1). Despite the induction of the modification machinery NisBTC only in the first phase, no impact on the modification efficiency was observed.^25,31^ Using this system, Deng et al. previously reported the incorporation of Hpg into nisin, albeit with a relative low yield and sometimes modest incorporation efficiency.^25^ Recently, we developed a new expression system for incorporating Met analogs, including azidohomoalanine (Aha), norleucine (Nle), and ethionine (Eth), into nisin, resulting in up to 8 times higher protein yield and complete replacement of Met by one of these Met analogs.^32^ However, the Met analog propargylglycine (Pra) was not well translated into nisin.^32^ Like Pra, the Met analog, Hpg (Figure 1f) contains an alkyne group which can react with an azido-sugar moiety using copper (Cu^+^) catalyzed azide–alkyne click chemistry (Figure 1g). In this work, the new expression system is used to incorporate Hpg in nisin and its variants cesin^28^ (Figure 1c) and rombocin^33^ (Figure 1d).

### 2.2 Production of Nisin Variants with Analog Incorporated

There are two Met residues in wild-type nisin (Figure 1e) and rombocin (Figure 1d), located at positions 17 and 21 in nisin and at positions 17 and 20 in rombocin. To avoid simultaneous analog incorporation at both sites, single-Met containing mutants were created. Previous studies revealed that nisin with a mutation at sites M17 and/or M21 could retain or even shown increased antimicrobial activity.^34^ Four single Met mutants were constructed, namely nisin­(M17I), nisin­(M21V), rombocin­(M17I), and rombocin­(M20V). The residues Ile or Val were chosen as substituents as their side chain hydrophilicities and sizes are quite similar to the Met side chain. The highest production yield was observed when Met was supplemented to nisin mutant nisin­(M21V), yielding 8.7 mg/L pure peptide, similar to the production yield of wild-type nisin (9.5 mg/L), using this system.^32^ When instead Hpg was supplemented, the yield dropped to 4.3 mg/L (Figure 2a). The production yield for nisin­(M17I) decreased a little bit (10%) compared with nisin­(M21V) labeled with Met. In the presence of Hpg, this production yield decreases 57%. Production yields of 3.7 mg/L, 3.6 mg/L and 3.7 mg/L were observed for cesin, rombocin­(M17I) and rombocin­(M20V), respectively, in the presence of Met. Supplemented with Hpg, the yields decreased to 1.3 mg/L, 1.3 mg/L and 1.7 mg/L, respectively (Figures 2a and S2). Together, a 2-to-2.8-time lower production yield is observed when Met is replaced by Hpg for the nisin, cesin and rombocin constructs studied in this work.

**Figure 2:**
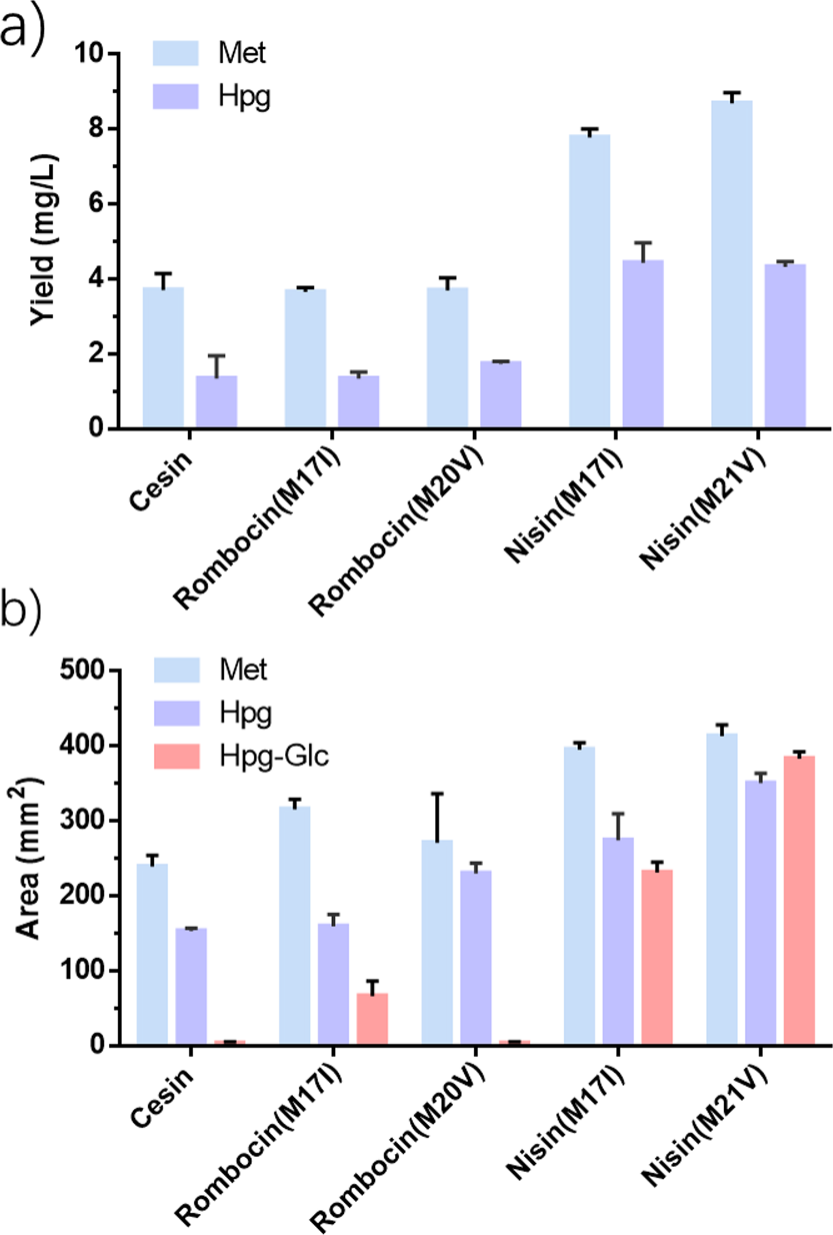
Production of single-Met containing nisin and nisin variants labeled with Met, Hpg or Hpg–Glc and their antibacterial activity against *E. faecium* strain. (a) Quantification of fully modified nisin variants labeled with Met or Hpg using HPLC as per Schmitt et al.^37^ (b) Evaluation of the antimicrobial efficacy of nisin and its variants against *E. faecium* through agar well diffusion assay. Each trial was repeated 3 times. The relative activity was determined by measuring inhibition zone diameters in millimeters, calculated as the area of the zone (π*r* ^2^) minus the area of the well (π*r* ^2^) in millimeters.

To assess the efficiency of post-translational modifications and incorporation of Hpg, all samples were further analyzed by mass spectrometry. In all cases, the incorporation of Hpg into nisin or its variants did not affect the dehydration efficiency, as fully dehydrated peptides (8 dehydrated residues for nisin, 7 for rombocin and 5 for cesin) were dominant (Table 1, Figure S3). The incorporation efficiency indicates the ratio between the amounts of peptides containing the analogs and the total amount of peptides. Met labeled peptides were undetectable, indicating the Hpg incorporation was always >99.5% (Table 1). In summary, the used new expression system^32^ results in the complete replacement of Met by Hpg and typically a multimilligram yield of peptide per liter is obtained, while post translational dehydration and ring formation processes are not negatively affected.

**Table 1:** The Molecular Mass and Incorporation Efficiency of the Nisin Variants

				observed mass (Da)		
peptide	modification	predicted mass (Da)		Met	Hpg	incorporation efficiency (%)
cesin	–5H_2_O	Met	2125.65^a^	2125.19		
		Hpg	2103.51^a^		2103.23	>99.5^c^
rombocin(M17I)	–7H_2_O	Met	4697.6^b^	4695.05		
		Hpg	4675.46^b^		4677.02	>99.5^c^
rombocin(M20V)	–7H_2_O	Met	4683.58^b^	4682.01		
		Hpg	4661.44^b^		4658.5	>99.5^c^
nisin(M17I)	–8H_2_O	Met	5669.75^b^	5668.4		
		Hpg	5647.61^b^		5645.37	>99.5^c^
nisin(M21V)	–8H_2_O	Met	5655.72^b^	5653.25		
		Hpg	5633.58^b^		5632.34	>99.5^c^

a The molecular mass of cesin following the removal of the leader segment by NisP.

b The molecular mass of the peptide including the leader segment but lacking the N-terminal Met. Normally, the initial Met of precursor is cleaved by the enzyme methionine aminopeptidase.

c >99.5% indicates that the peak of peptides containing Met is not detectable.

### 2.3 Antimicrobial Activity of Analog-Containing and Sugar-Clickable Derivatives

All nisin variants, either containing Met or the analog Hpg, were tested against Gram-positive pathogenic strain *Enterococcus faecium*. The results indicated that nisin­(M21V) exhibited the highest antibacterial activity and labeling with Hpg resulted in a 17% decrease in the zone of inhibition in agar well diffusion assays (Figure 2b). Incorporating Hpg into nisin­(M17I) also led to a decrease in activity by 30% compared to Met substitution. These findings are consistent with previous reports by Deng et al., who noted decreases in activity against *E. faecium* with Hpg-incorporated nisin­(M21V) and nisin­(M17I).^25^ The incorporation of Hpg had also a negative impact on the rombocin­(M17I) and rombocin­(M20V) mutants, resulting in a decrease in activity by 49% and 13%, respectively. When Hpg was incorporated into cesin, it led to a 36% reduction in activity.

Due to the high potency of the nisin mutants (Figure 2b), Hpg-incorporated nisin­(M17I) and nisin­(M21V) were purified (Figure S4) by HPLC and subjected to minimum inhibitory concentration (MIC) testing^35^ against four Gram-positive pathogenic strains, including *Staphylococcus aureus*, *Listeria monocytogenes*, *E. faecium* and *Bacillus cereus*. The results, presented in Table 2, show that Hpg incorporation into nisin­(M21V) had a negative impact on its activity against the aforementioned pathogenic strains, resulting in a 2-fold higher MIC. Hpg incorporation significantly influenced the antibacterial activity of nisin­(M17I), leading to a 4-fold decrease against *S. aureus* and *E. faecium*, and an 8-fold decrease against *L. monocytogenes* and *B. cereus*. Previous results highlighted the importance of the hinge region (NMK, positions 20 to 22) for nisin activity,^34,36^ and our results demonstrate that already changing several atoms in the Met side chain have a large effect on the activity (Figure 1f).

**Table 2:** Antimicrobial Profile of Nisin and Hpg-Labeled Nisin Mutants against Selected Gram-Positive Strains

	MIC (μg/mL)		
organism and type	nisin	nisin(M17I)(Hpg)	nisin(M21V)(Hpg)
Staphylococcus aureus LMG15975 (MRSA)^a^	7.6	30.4	15.2
*Listeria monocytogenes* LMG10470	15.2	121.5	30.4
*Enterococcus faecium* LMG16003 (VRE)^b^	3.8	15.2	7.6
*Bacillus cereus* CH-85	15.2	121.5	30.4

a MRSA, methicillin-resistant *Staphylococcus aureus*.

b VRE, vancomycin-resistant enterococci.

Hpg-incorporated peptides were used in click chemistry reactions with 2-azido-2-deoxy-d-glucose and the antibacterial activity against *E. faecium* strain was evaluated (Figure 2b). After functionalizing with a glucose moiety, the activity of the conjugate nisin­(M21V)–Glc improved by 9% compared to when no sugar was attached, becoming slightly lower (5%) than nisin­(M21V) labeled with Met. On the other hand, when sugar was attached at the hinge region (nisin­(M17I)), the activity decreased 15% compared to Hpg-nisin­(M17I) and 41% compared to nisin­(M17I). In the case of the cesin and rombocin variants, sugar attachment also led to a decrease in activity. Following sugar attachment, both cesin and rombocin­(M20V) exhibited a complete loss of activity at the tested peptide concentrations. Rombocin­(M17I)–Glc showed a 80% decrease compared to rombocin­(M17I).

Due to the potent activity of nisin­(M21V) clicked with glucose, nisin­(M21V) was selected for further studies, purified by HPLC (Figures S5 and S6) and click chemistry reactions were performed with 2-azido-2-deoxy-d-glucose (yielding nisin­(M21V)–Glc) and 6-azido-6-deoxy-d-galactose (yielding nisin­(M21V)–Gal) (Figure 3a). Compared to wild-type nisin or Hpg-labeled nisin­(M21V), nisin­(M21V)–Glc and nisin­(M21V)–Gal exhibited reduced activity against two tested pathogenic strains, *S. aureus* and *B. cereus*, and significantly lower activity against *Enterococcus faecalis*. However, these two sugar modified nisin variants retained high potency against *E. faecium*, with the galactose-attached variant showing slightly higher activity compared to the glucose-attached counterpart (Figure 3b). Next, the MIC values of nisin­(M21V)–Gal against four Gram-positive pathogenic strains were determined. Nisin­(M21V)–Gal displayed potent activity against *E. faecium* at a low concentration of 3.8 μg/mL, similar to that of nisin. However, nisin­(M21V)–Gal exhibited 2 times higher MIC values against *S. aureus* and *B. cereus*, and 4 times higher MIC values against *E. faecalis* compared to nisin (Figure 3c). Overall, the newly developed compound nisin­(M21V)–Gal, modified at residue position 17 of nisin with an attached galactose moiety, demonstrates highly specific and potent antibacterial activity against a vancomycin resistant *E. faecium* strain.

**Figure 3:**
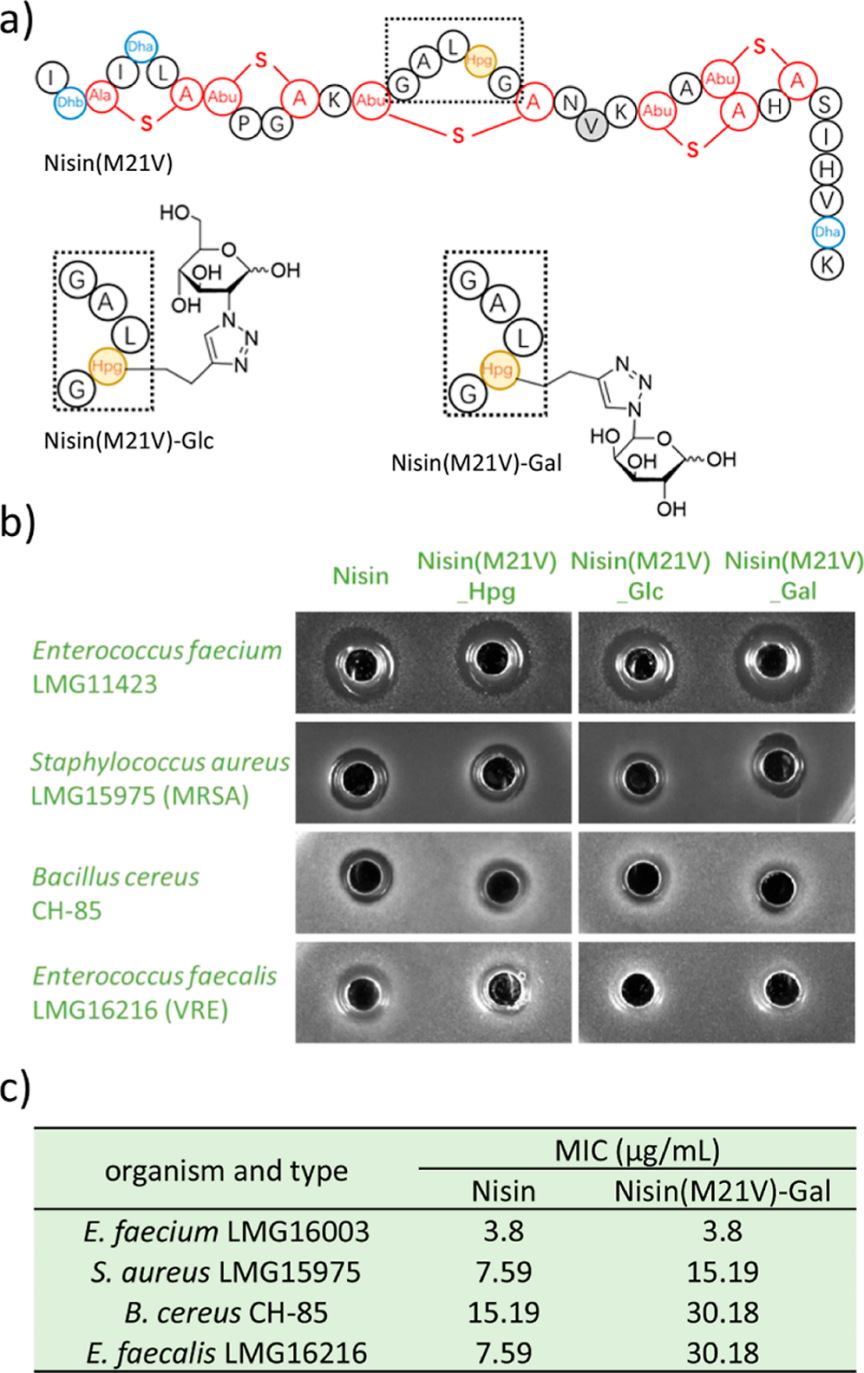
Antibacterial activity of nisin and nisin­(M21V) variants. (a) The structure of nisin­(M21V), nisin­(M21V)–Glc and nisin­(M21V)–Gal. (b) Agar well diffusion assay to test the HPLC-purified nisin and nisin mutants against four pathogenic Gram-positive bacteria. Whereas wild-type nisin exhibits broad activity against all tested strains, the glycosylated nisin variants demonstrate selective efficacy. (c) MIC values of HPLC-purified nisin and nisin­(M21V)–Gal tested against four pathogenic Gram-positive bacteria.

### 2.4 The Solubility of Nisin­(M21V)–Gal Is 3.9 Times Higher Compared to Wild-Type Nisin

The solubility of nisin at ambient temperature and neutral pH is 1–2 mg/mL^38^ and this low solubility is a limiting factor in its applications, particularly in clinical settings.^4,39^ To enhance the nisin solubility at neutral pH, Rollema et al. introduced an extra lysine residue and this resulted in a 4 to 7 fold higher solubility.^38^ Highest solubility at pH 7 was obtained for nisin Z mutant M31K (7–8 mg/mL). The covalent modification of a therapeutic peptide or protein with sugar units is an established strategy to enhance drug solubility and bioavailability. The N-terminal segment of nisin contains a relatively high proportion of hydrophobic residues, while the C-terminal portion is more hydrophilic, featuring positively charged lysine and histidine side chains (Figure 1e). Attaching a hydrophilic sugar moiety to the C-terminal region of nisin therefore may not substantially alter its amphiphilic nature. To assess the solubility of nisin and its new variants at neutral pH, wild-type nisin or nisin­(M21V)–Gal were added to a 50 mM PBS buffer solution (pH 7.2) at room temperature until precipitation occurred (Figure S7a). After equilibration, the concentration of dissolved nisin in the saturated solution was determined using high-performance liquid chromatography (HPLC). For this, a standard nisin concentration curve (Figure S7b) was created by dissolving commercially available nisin at pH 4, as nisin exhibits greater solubility at acidic pH levels. As expected at neutral pH, nisin shows low solubility (2.2 mg/mL), but with the attachment of galactose, the solubility significantly increased to 8.6 mg/mL (Figure 4). Together, this work demonstrates that covalent attachment of a sugar moiety at nisin can narrow its activity spectrum and favorably contributes to its solubility at neutral pH.

**Figure 4:**
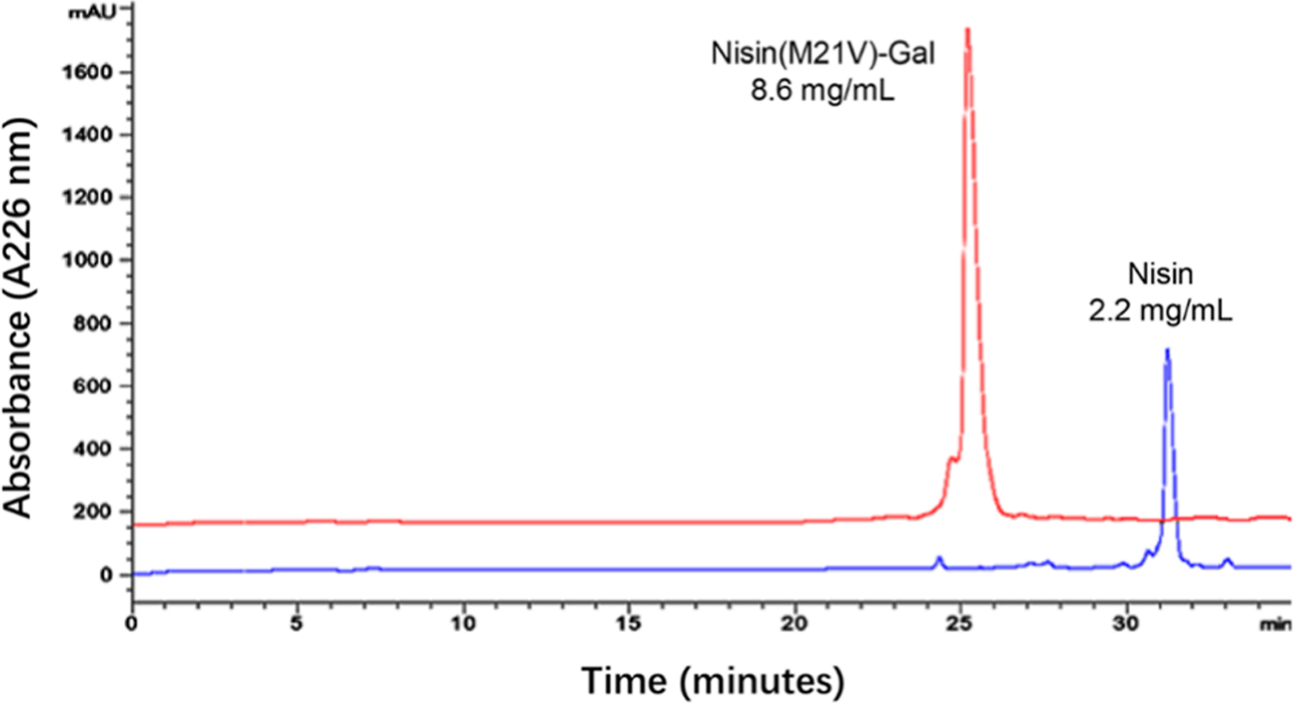
Solubility of nisin and nisin­(M21V)–Gal at neutral pH. The saturated solutions were analyzed using HPLC with an absorbance wavelength of 226 nm.

### 2.5 Nisin­(M21V)–Gal Binds to Cell Wall Synthesis Precursor Lipid II

The antimicrobial activity of nisin is due to creating pores in the membrane of the target microbe and hindering its cell wall synthesis through specific binding to lipid II, a critical precursor in peptidoglycan biosynthesis.^6^ To explore the impact of the attached sugar group in nisin on its mode of action, we examined its binding capacity to lipid II. The addition of externally purified lipid II resulted in a reduction in the antimicrobial activity of both nisin and nisin­(M21V)–Gal against *E. faecium*, disrupting the typical circular halo induced by antibiotics (Figure 5a). In contrast, the nonlipid II-binding antibiotic daptomycin maintained its antimicrobial efficacy against the tested strains even after the introduction of purified lipid II, leading to a circular halo (Figure 5a). Nisin binds lipid II through the lipid binding domain formed by rings A and B. In nisin­(M21V)–Gal, the sugar is attached to ring C at position 17, leaving rings A and B unaltered. The results show that despite undergoing modifications, nisin­(M21V)–Gal maintained its capacity to bind to lipid II, similar to that of nisin.

**Figure 5:**
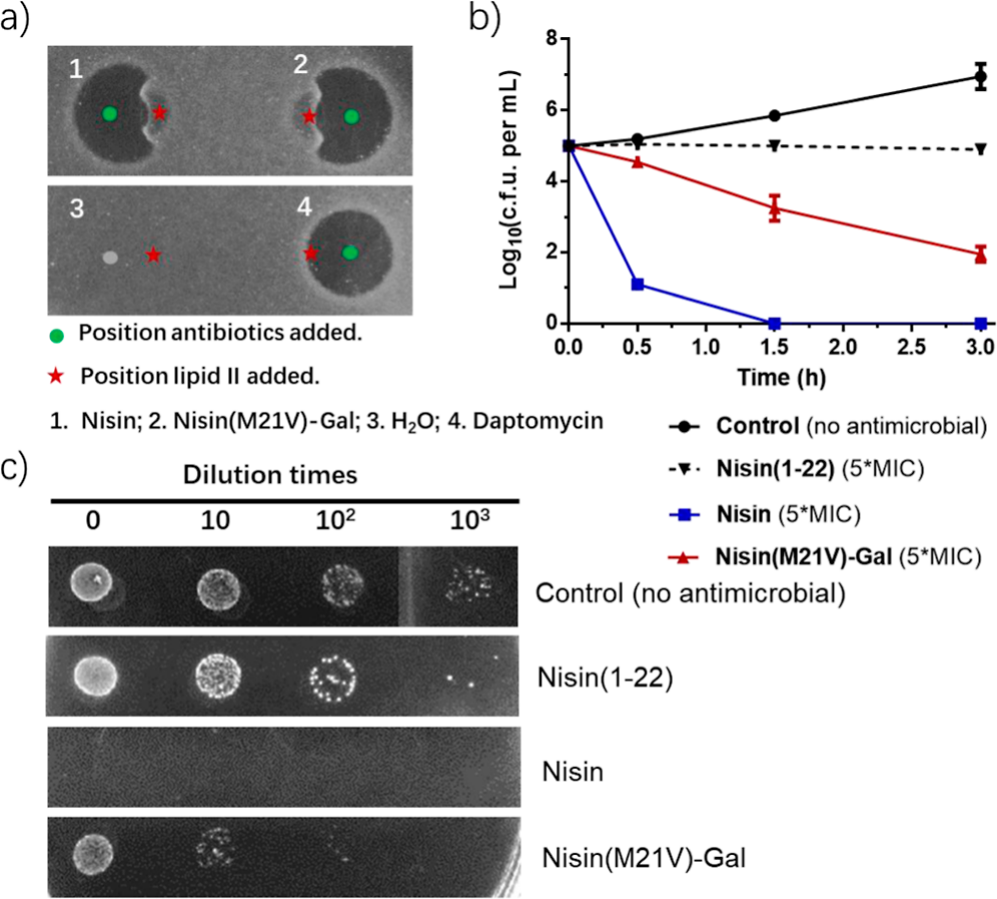
Lipid II binding and bactericidal activity of nisin­(M21V)–Gal. (a) Spot-on-lawn assay assessing the binding of nisin­(M21V)–Gal to the cell wall synthesis precursor lipid II. Nisin served as positive control, while daptomycin and H_2_O were the negative controls. (b) Time-dependent killing assay to evaluate the bactericidal activity of nisin­(M21V)–Gal. Lantibiotics at a 5-fold MIC concentration were tested against *E. faecium*, with nisin as the bactericidal control and nisin(1–22) as the bacteriostatic control. The experiment was replicated 3 times, and standard deviation (SD) was calculated. (c) Examination of *E. faecium* cells treated with lantibiotics at a 5-fold MIC concentration after 3 h of incubation. The dilution factors of the solutions are indicated at the top of the figure, with 5 μL of solutions spotted on the plates.

### 2.6 Nisin­(M21V)–Gal Exhibits Bactericidal Activity, Albeit at a Slower Rate Compared to Nisin

Nisin can create pores in the target cell membrane through its C-terminal domain. A truncated variant of nisin, nisin(1–22), demonstrates a bacteriostatic effect by only being able to bind to lipid II, thereby halting cell growth without causing cell death.^40^ Time-dependent pathogen killing studies with nisin­(M21V)–Gal, nisin(1–22), and nisin against *E. faecium* cells were conducted to determine the bacteriostatic or bactericidal nature of antimicrobials (Figure 5b,c). The results showed that nisin (M21V)–Gal achieved a thousand-fold reduction in bacterial population after 3 h of incubation, indicating its ability not only to inhibit cell division like nisin(1–22) but also to decrease the number of viable bacterial cells (Figure 5b). In contrast, nisin exhibited a faster action than nisin­(M21V)–Gal, leading to a significant reduction in viable cell population with complete cell eradication after 1.5 h (Figure 5b). Our findings suggest that nisin­(M21V)–Gal displays bactericidal activity against bacterial cells, but the rate of cell death is slower compared to nisin.

### 2.7 Nisin­(M21V)–Gal Lost Its Pore-Forming Ability

The pore-forming abilities of nisin­(M21V)–Gal and nisin were investigated using potassium ion release experiments with the potassium ion-sensitive fluorescent probe PBFI.^41^ Nisin elicited an immediate signal increase at various peptide concentrations (Figure 6a) after antibiotic added (Figure 6b), indicating the release of intracellular potassium ions, with higher peptide concentrations leading to more potassium ion release (Figure 6b). In contrast, nisin­(M21V)–Gal did not demonstrate this effect, even at a peptide concentration of 32 times MIC. Additionally, we examined the membrane potential of *E. faecium* cells treated with nisin­(M21V)–Gal using the membrane potential-sensitive fluorescent probe DiSC3(5).^42^ The results presented in Figure 6c indicate that nisin­(M21V)–Gal also lost the ability to induce membrane depolarization. Our findings suggest that the attachment of a sugar moiety at residue position 17 in ring C of nisin reduces its typical pore-forming ability, explaining its decreased bactericidal activity against certain pathogens.

**Figure 6:**
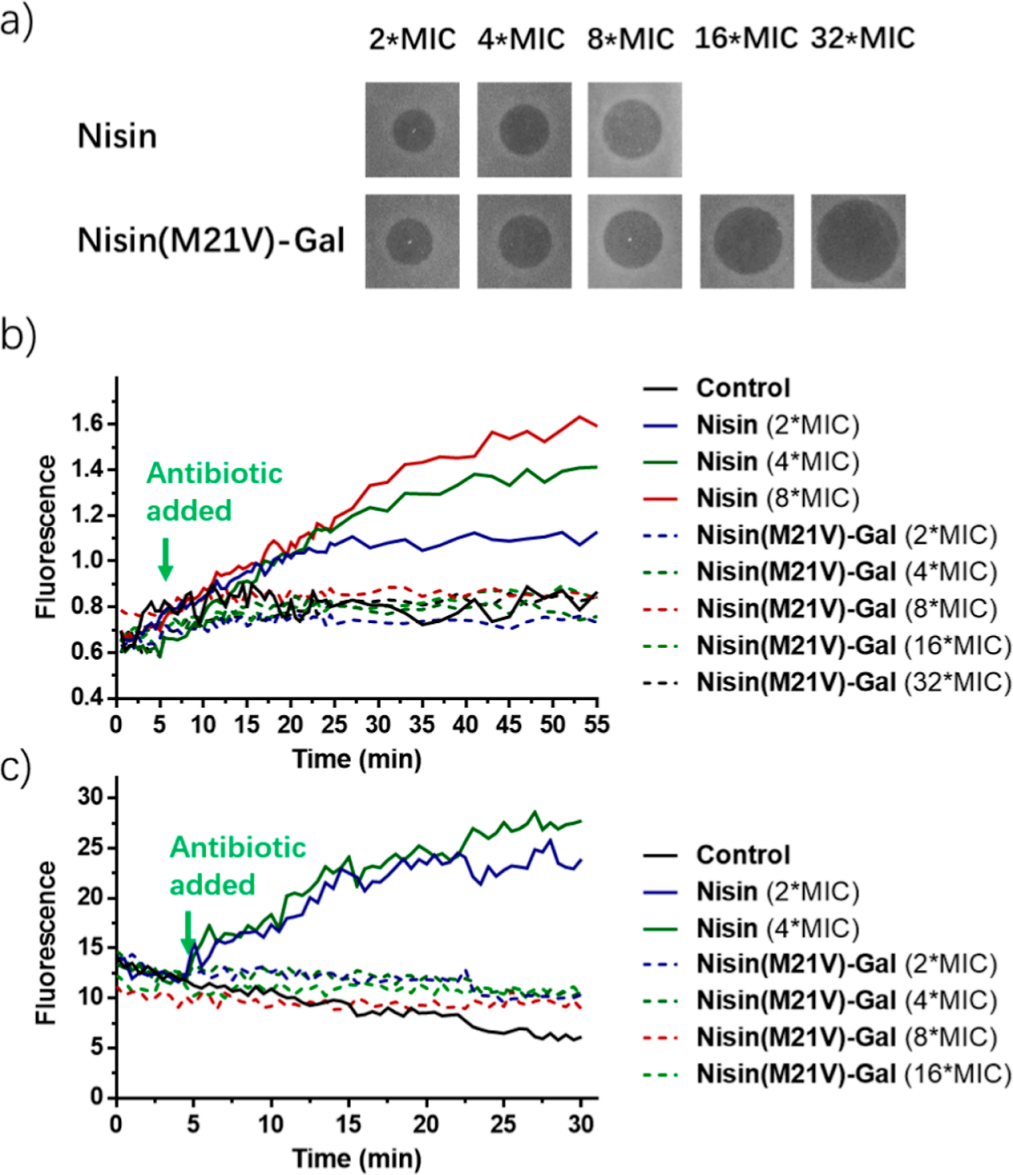
Impact of nisin and nisin­(M21V)–Gal on *E. faecium*. (a) Spot-on-lawn assay evaluating the effects of different concentrations of nisin and nisin­(M21V)–Gal on *E. faecium*. (b) Assessment of potassium leakage using the increase in fluorescence of the PBFI probe after the introduction of varying concentrations of antimicrobials at *T* = 5 min. (c) Monitoring changes in membrane potential of *E. faecium* through the increase in fluorescence of the DiSC3(5) probe following treatment with different concentrations of antimicrobial agents added at *T* = 5 min. All experiments were performed in triplicate, and a representative image is presented.

The exact role of ring C remains unclear; however, it has been shown to be critical for nisin’s biological activity.^4^ For instance, converting the thioether bond of ring C into a disulfide bond resulted in a dramatic reduction in antimicrobial activity.^43^ Mutagenesis studies on residue M17 have demonstrated its influence on antimicrobial activity: the M17Q mutation enhanced effectiveness against *Staphylococcus epidermidis*,^44^ whereas M17W^31^ and M17I^25,32^ mutations reduced activity against various Gram-positive bacteria. Recently, we introduced the Met analog Aha at position 17 and used click chemistry to modify it with hydrophobic tail molecules resulting in constructs showing altered bioactivity.^45^ Modification with a tail containing a benzyl group yielded to one of the most active constructs and this construct binds lipid II and forms pores in *S. aureus* and *E. faecium*. In summary, this and previous studies show engineering of residue 17 is a powerful means to alter the antimicrobial spectrum of nisin. Potent activity can be obtained even when the engineering results in the loss of pore forming activity, as shown in this study for nisin­(M21V)–Gal.

## 3 Conclusion

Engineering of therapeutic peptides and proteins via glycosylation has yielded exciting results as important properties like solubility and thermo- and proteolytic-stability could be significantly improved.^23^ The protocols presented in this work make it possible to explore this route for RiPPs. First results, obtained for nisin and two of its variants, are very encouraging. Met could be completely replaced by Hpg, yielding typically multi mg yield of the peptide per liter culture. The orthogonal click chemistry reaction allowed to site specifically label the peptide with an azido sugar. One sugar labeled nisin variant presented in this work features potent strain specific antimicrobial activity and enhanced solubility. The protocols presented in this work expands the toolkit for improving and discovering (lanthi)­peptide-based drugs and can contribute to tackle hurdles for making them suitable for clinical applications.

## 4 Materials and Methods

### 4.1 Materials

The reagents utilized in molecular biology experiments were obtained from Thermo Fisher Scientific (Waltham, MA). Unless otherwise noted, all chemicals were acquired from Sigma-Aldrich (St. Louis, MO). The Met analog l-homopropargylglycine (Hpg) was obtained from Lumiprobe Corporation (Maryland, Americas). 2-Azido-2-deoxy-d-glucose (CAS no. 56883-39-7) and 6-azido-6-deoxy-d-galactose (CAS no. 66927-03-5) were purchased from Sigma-Aldrich.

### 4.2 Bacterial Strains, Plasmids, and Growth Conditions

The bacterial strains and plasmids used in this study are listed in Table S1. All *L. lactis* strains were grown in M17 broth (BD Difco) supplemented with 0.5% (w/v) glucose (GM17) at 30 °C. When appropriate, 5 μg/mL chloramphenicol (Cm) and/or erythromycin (Em) were added to the media. *L. lactis* NZ9000 was used as the host for cloning, plasmid maintenance, and peptide expression. Chemical defined medium lacking tryptone (CDM-P)^37^ was used for peptide expression and Met analog incorporation.

### 4.3 Molecular Biology Techniques

The PCR primers used in this study, all purchased from Biolegio B.V. (Nijmegen, The Netherlands), are listed in Table S2. To construct plasmids encoding the mutations, the template plasmid was amplified using a phosphorylated downstream sense (or upstream antisense) primer in conjunction with an upstream antisense (or downstream sense) primer. Amplification was performed using Phusion High-Fidelity DNA polymerase (Thermo Fisher Scientific). Following agarose gel electrophoresis (1%), PCR products were validated, and the correct molecular weight band was isolated and purified using the NucleoSpin Gel and PCR Cleanup Kit (Bioke, Leiden, The Netherlands). Subsequent self-ligation of the DNA fragment was carried out with T4 DNA ligase. The ligation product was desalted and then transformed into *L. lactis* NZ9000 following established protocols^46^ with a Bio-Rad Gene Pulser (Bio-Rad, Richmond, CA). The plasmid was isolated and confirmed by sequencing using the pNZ-f primer (Table S2).

### 4.4 Expression of Hpg-Incorporated Peptides

To confirm successful incorporation of Hpg, a small-scale (20 mL) expression and purification process was carried out. *L. lactis* NZ9000 cells containing the *nisBTC* plasmid were subjected to electroporation with the *nisA* gene-harboring plasmid (100 ng), then plated on GM17 agar plates supplemented with chloramphenicol (5 μg/mL) and erythromycin (5 μg/mL) for overnight incubation at 30 °C. Following this, a single colony was selected and transferred to 4 mL of GM17CmEm medium for growth. Subsequently, 0.5 mL of the overnight culture was diluted in 20 mL of the same medium. Upon reaching an OD_600_ of approximately 0.4, 0.5 mM ZnSO_4_ was introduced to induce the expression of the nisin modification machinery NisBTC. After 3 h, the cells underwent three washes with phosphate-buffered saline (pH 7.2) and were then resuspended in 20 mL of CDM-P devoid of Met. Following a 1 h starvation period, Met (38 mg/L) or the Met analog Hpg (50 mg/L), along with 10 ng/mL nisin, were added to induce peptide expression. Following overnight growth, the supernatant was obtained by centrifugation at 8000*g* for 15 min. The peptides were then precipitated with 10% trichloroacetic acid (TCA) on ice for a minimum of 2 h, followed by centrifugation at 10,000*g* and 4 °C for 45 min. The resulting pellets were washed with 10 mL of ice-cold acetone to eliminate TCA. Subsequently, the samples were dried in a fume hood and stored at −20 °C or resuspended in 0.2 mL of a 0.05% aqueous acetic acid solution for further analysis.

### 4.5 Tricine-SDS-PAGE Analysis

The peptides were analyzed by the Tricine-SDS-PAGE gel as described by Schagger.^47^ 10 μL of sample was mixed with 2 μL loading dye and applied to a 16% gel. Coomassie brilliant blue G-250 was used for protein staining.

### 4.6 Mass Spectrometry

A volume of 1 μL of the peptide was spotted onto the target, dried, and rinsed multiple times with Milli-Q water. Subsequently, an equivalent volume of matrix solution (5 mg/mL α-cyano-4-hydroxycinnamic acid dissolved in 50% acetonitrile with 0.1% trifluoroacetic acid) was applied on top of the sample. Mass spectra were acquired using an Applied Biosystems 4800 Plus matrix-assisted laser desorption/ionization time-of-flight analyzer (MALDI-TOF) operating in linear mode with external calibration. The analog incorporation efficiency was determined by assessing the peak areas of the analog-containing peptides and the Met-containing peptides.

### 4.7 Agar Well Diffusion Assay

An overnight culture was added at a concentration of 0.1% (v/v) to molten GM17 agar (for *E. faecium* and *E. faecalis*) or LB agar (for *S. aureus* and *B. cereus*) at 45 °C, and then 30 mL of this mixture was poured onto a plate. After the agar solidified, wells of 8 mm were created by punching in the agar and filled with 30 μL of a 1 mg/mL lantibiotic solution. If needed, lantibiotics were activated by adding 3 μL of 1 mg/mL NisP^48^ directly to the well. The quantity of nisin was determined using HPLC as previously outlined.^37^ The agar plate was then incubated at 37 °C overnight, and the zones of inhibition were measured. The presented data results from three independent experiments. Zone diameters were measured in millimeters and recorded as the area of the zone (π*r* ^2^) minus the area of the well (π*r* ^2^) in millimeters.

### 4.8 Purification of Nisin Variants Labeled with Hpg

To produce larger quantities of nisin variants, experiments were conducted on a 2 L scale. The supernatant pH was adjusted to 7.0 and then incubated with purified NisP at 37 °C for 3–6 h to remove the leader sequence. Subsequently, the treated supernatant was passed through a C_18_ open column (Spherical C_18_, 5 g, particle size: 40–75 μm, Sigma-Aldrich). The column was washed with 40 mL of varying concentrations (25%, 30%, 40%, and 60%) of buffer B (buffer A: distilled water with 0.1% TFA; buffer B: acetonitrile with 0.1% TFA). The active fractions were then freeze-dried and subjected to further purification using an Agilent 1200 series HPLC system equipped with a C_12_ column (Jupiter 4 μm Proteo 90Å, 250 × 4.6 mm, Phenomenex). The eluted peak exhibiting activity and the correct molecular weight was collected, lyophilized, and stored at 4 °C until required for subsequent use.

### 4.9 Minimal Inhibitory Concentration Assay

The minimal inhibitory concentration (MIC) values were assessed through broth microdilution following standard guidelines.^35^ The inoculum was adjusted to around 5 × 10^5^ CFU/mL. MIC was characterized as the lowest concentration of the antimicrobial substance at which no visible growth was observed following overnight incubation at 37 °C.

### 4.10 Glycosylation of Hpg-Labeled Nisin Variants

Stock solutions of CuSO_4_ (100 mM), sodium ascorbate (1 M), and BTTAA (2-(4-((bis­((1-*tert*-butyl-1*H*-1,2,3-triazol-4-yl)­methyl)­amino)­methyl)-1*H*-1,2,3-triazol-1-yl)­acetic acid, 50 mM) were prepared. Nisin variants labeled with Hpg (100 μg) were dissolved in 100 mM phosphate buffer (pH 7.0, final reaction volume: 200 μL), and 5 equiv of azido-sugar were added to the solution. Subsequently, a premix of CuSO_4_ (4 μL) and BTTAA (40 μL) stock solutions was added, followed by the addition of 20 μL sodium ascorbate. The reaction was conducted at 37 °C and after 1 h the reaction was quenched with 3 mL buffer (H_2_O/acetonitrile, 95:5 + 0.1% TFA) and the modified peptide purified through HPLC. The isolated yield of the clicked peptide ranged from 65% to 80%.

### 4.11 Solubility Studies

Wild-type nisin (product code: 0305, Handary, Belgium) or nisin­(M21V)–Gal was added to a 50 mM PBS buffer solution until precipitation occurred. Equilibrium was established by continuous shaking for 30 min, followed by centrifugation for 3 min at 15,000*g*. The concentration of the saturated nisin solution was determined using HPLC. For this, a nisin concentration curve was created, for which different amounts of nisin were dissolved in a 0.05% acetic acid solution at pH 4, with concentrations ranging from 0.2 mg/mL to 7 mg/mL. All experiments were carried out at room temperature and were repeated 3 times.

### 4.12 Spot-On-Lawn Assay to Measure Peptide–Lipid II Complex Formation

To assess the interaction between the peptide and lipid II, an overnight culture of *E. faecium* was added to 0.8% GM17 (w/v) at 45 °C with a final concentration of 0.1% (v/v), and the mixture was then poured into 10 mL plates. The binding of the peptide to lipid II was further examined by spotting purified lipid II (300 μM, 2 μL) at the periphery of the antibiotic inhibition zone. In brief, antimicrobials were applied to the agar plate. Once the antimicrobial solution drops had dried, lipid II was spotted at the edge of the inhibition zone. After the drops had dried, the plates were incubated overnight at 37 °C.

### 4.13 Time-Kill Assay

The antimicrobial effectiveness of nisin, nisin(1–22), and nisin­(M21V)–Gal was assessed using a method previously described by Guo et al.^30,49^ In summary, an overnight culture of *E. faecium* was diluted 50 times in GM17 medium and cultured at 37 °C. The bacteria were grown until reaching an optical density at 600 nm (OD_600_) of 0.5, and then the cell concentration was adjusted to 5 × 10^5^ colony-forming units per milliliter (CFU/mL). Following this, the bacteria were exposed to a 5-fold MIC of each peptide. An untreated cell suspension was used as a control. At specific time intervals, 50 μL samples were taken, and both undiluted and 10-fold serially diluted suspensions were plated on GM17 agar. The plates were then incubated overnight at 37 °C, and the resulting colonies were counted and expressed as CFU/mL. Each experiment was conducted in triplicate.

### 4.14 Potassium Ion Efflux Assays

To conduct the K^+^ release assay, the K^+^-specific fluorescent probe PBFI was utilized. *E. faecium* was cultured in GM17 medium until it reached an OD_600_ of 0.6, at which point the cells were harvested (5000*g*, 5 min) and washed twice with 10 mM HEPES (pH 7.2) containing 0.5% glucose. The cells were then suspended in the same buffer supplemented with 10 μM PBFI. Data analysis was carried out using a Varioskan LUX Multimode Microplate Reader (Thermo Fisher Scientific), with cell excitation at 346 nm and fluorescence emission measurement at 505 nm to establish a baseline signal before the introduction of varying concentrations of antibiotics. Nisin was utilized as the positive control in this study.

### 4.15 Determination of Membrane Potential

To assess the membrane potential, the membrane potential-sensitive fluorescent dye DiSC3(5) was employed. *E. faecium* was grown to an OD_600_ of 0.8, then centrifuged at 5000*g* for 5 min and washed twice in 10 mM HEPES with 10 mM glucose (pH 7.2). The cell density was adjusted to an OD_600_ of 0.2 and loaded with 2 μM DiSC3(5) dye, followed by a 20 min incubation in darkness to stabilize the probe fluorescence. Subsequently, the cell suspension was transferred to a 96-well microplate and incubated for 5 min with 100 mM KCl. Antibiotics were then introduced at a varied concentration, and the fluorescence was monitored for 25 min. The excitation and emission wavelengths on the fluorescence spectrometer were set to 622 and 670 nm, respectively. Three replicates were conducted, and a representative example is presented.

## Supplementary Material



## Data Availability

All data supporting the findings of this study are available within the paper and its Supporting Information file.

## Notes

Schematic representation of the nisin biosynthetic pathway and the force-feeding method for incorporating ncAA; Tricine-SDS-PAGE analysis of nisin variants cultured in Met or Hpg; MALDI-TOF MS analysis of TCA precipitated peptides with Met or Hpg incorporated; MALDI-TOF MS analysis of HPLC-purified nisin mutants labeled with Hpg; MALDI-TOF MS analysis of HPLC-purified nisin­(M21V) with attached sugar moiety; HRLC-MS/MS analysis of HPLC-purified nisin­(M21V)–Gal; standard nisin concentration curve; plasmids and strains used in this study; primers used in this study (PDF)
