# Supplementary material for: Functionalizing Nisin with a Sugar Moiety Improves Its Solubility and Results in an Altered Antibacterial Spectrum and Mode of Action
Source: ACS Synth Biol. 2025 Aug 12;14(9):3568–77. doi: 10.1021/acssynbio.5c00353 (PMC12455637; doi:10.1021/acssynbio.5c00353)
Supplement: Supplementary file 1 [file sb5c00353_si_001.pdf]

Supporting Information for

Functionalizing nisin with a sugar moiety improves its solubility and results in an altered antibacterial spectrum and mode of action

Longcheng Guo[a, b] , Oscar P. Kuipers[a], Jaap Broos[a,#]

[a] Department of Molecular Genetics, Groningen Biomolecular Sciences and Biotechnology Institute, University of Groningen, Groningen, 9747 AG, The Netherlands

[b] Present address: Department of Chemistry, The University of Hong Kong, Pokfulam Road, Hong Kong, China

[#] Correspondence to Jaap Broos, j.broos@rug.nl

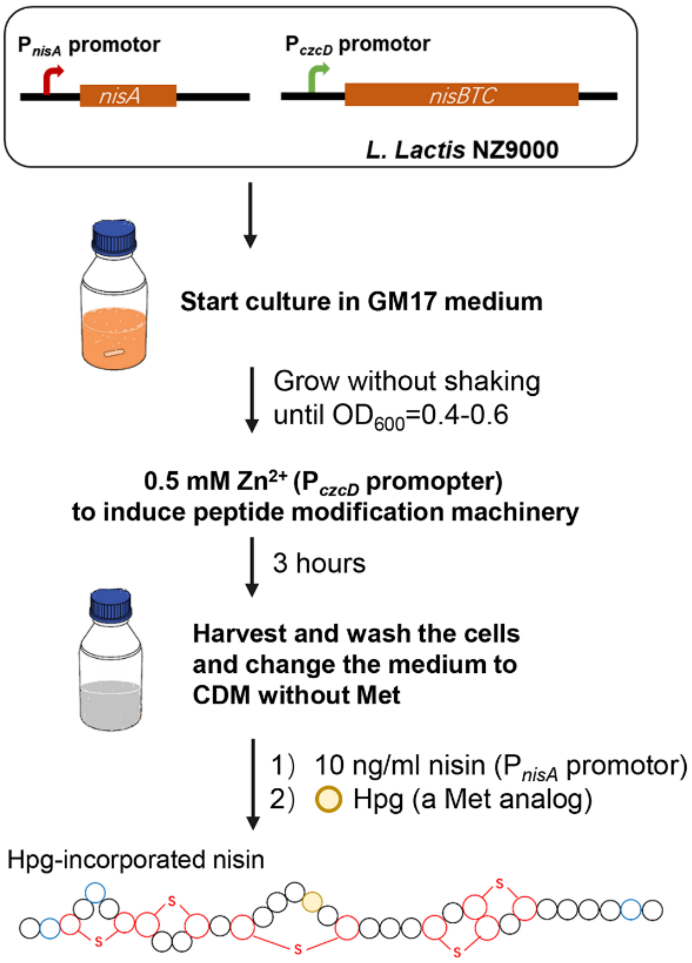

Figure S1 Schematic representation of the nisin biosynthetic pathway and the force-feeding method for incorporating a non-canonical amino acid (ncAA) in nisin. The expression medium initially contains all canonical amino acids (cAAs). Upon reaching an OD_{600} value of 0.4-0.6, 0.5 mM Zn^{2+} is introduced to trigger the peptide modification machinery NisBTC. After 3 hours, the cells are harvested, washed thrice with PBS (pH 7.2), and then resuspended in a synthetic medium lacking methionine but containing the methionine analog Hpg. Nisin (10 ng/mL) and analog are added to induce the expression of the peptide incorporating Hpg.

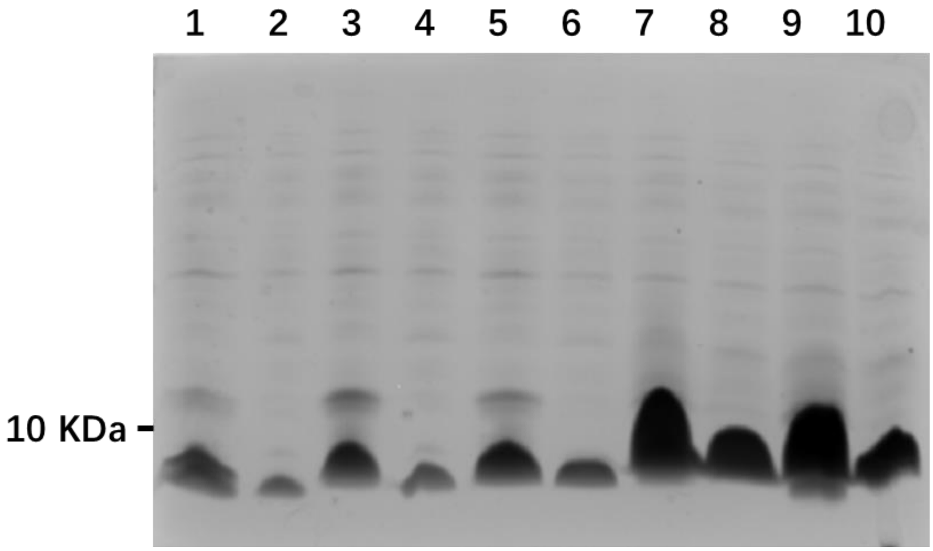

Figure S2 Tricine-SDS-PAGE analysis showing the expression of nisin variants in the presence of Met or Met analog Homopropargylglycine (Hpg). Each lane represents peptide extracted from 1 ml supernatant. Lane **1**: Cesin (Met), Lane **2**: Cesin (Hpg), Lane **3**: Rombocin(M17I)(Met), Lane **4**: Rombocin(M17I)(Hpg), Lane **5**: Rombocin(M20V)(Met), Lane **6**: Rombocin(M20V)(Hpg), Lane **7**: Nisin(M17I)(Met), Lane **8**: Nisin(M17I)(Hpg), Lane **9**: Nisin(M21V)(Met), Lane **10**: Nisin(M21V)(Hpg).

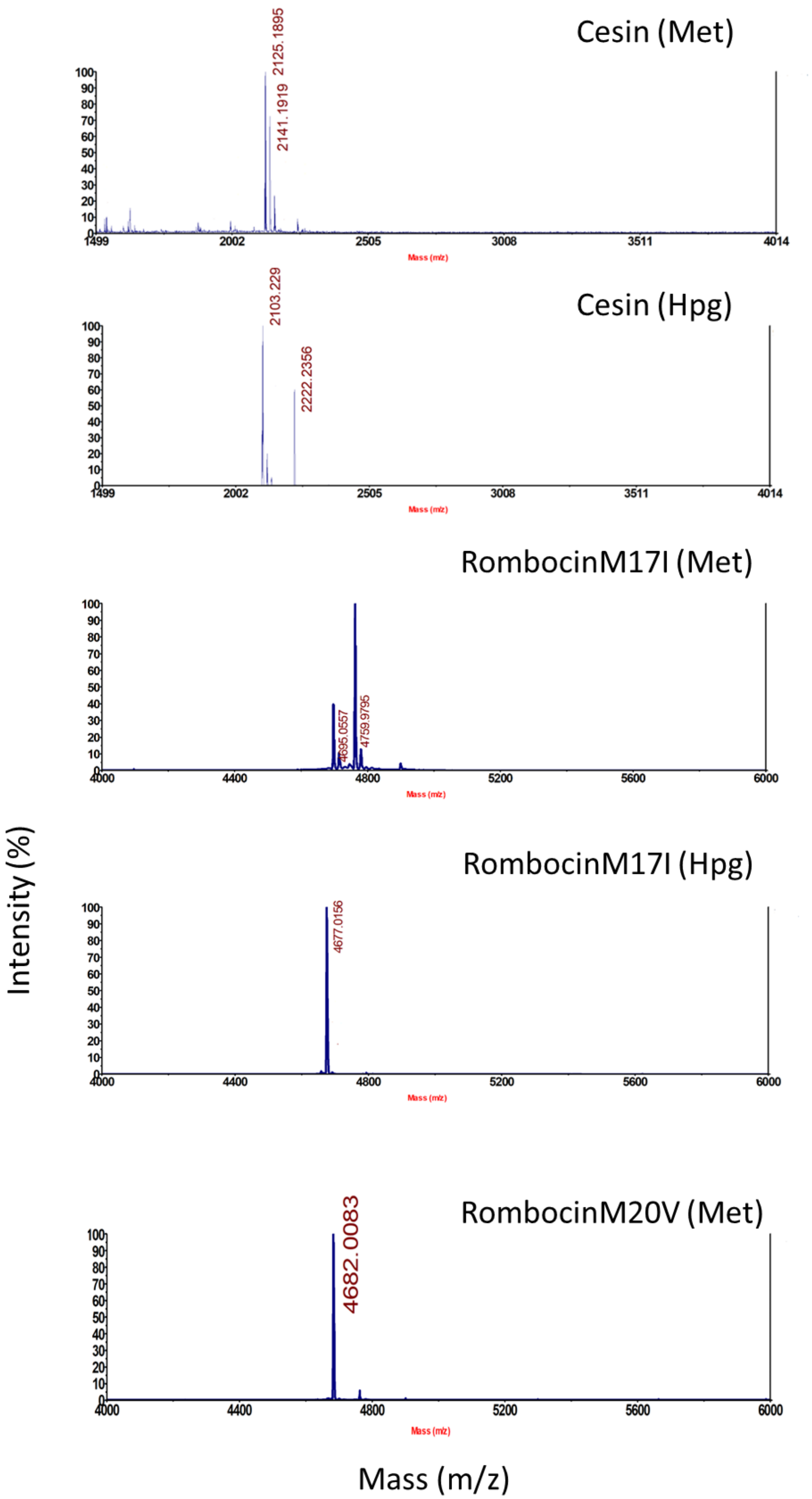
Cesin (Met)
2125.1895
2141.1919
Cesin (Hpg)
2103.229
2222.2356
RombocinM17I (Met)
4695.0557
4759.9795
RombocinM17I (Hpg)
4677.0156
RombocinM20V (Met)
4682.0083
Intensity (%)
Mass (m/z)

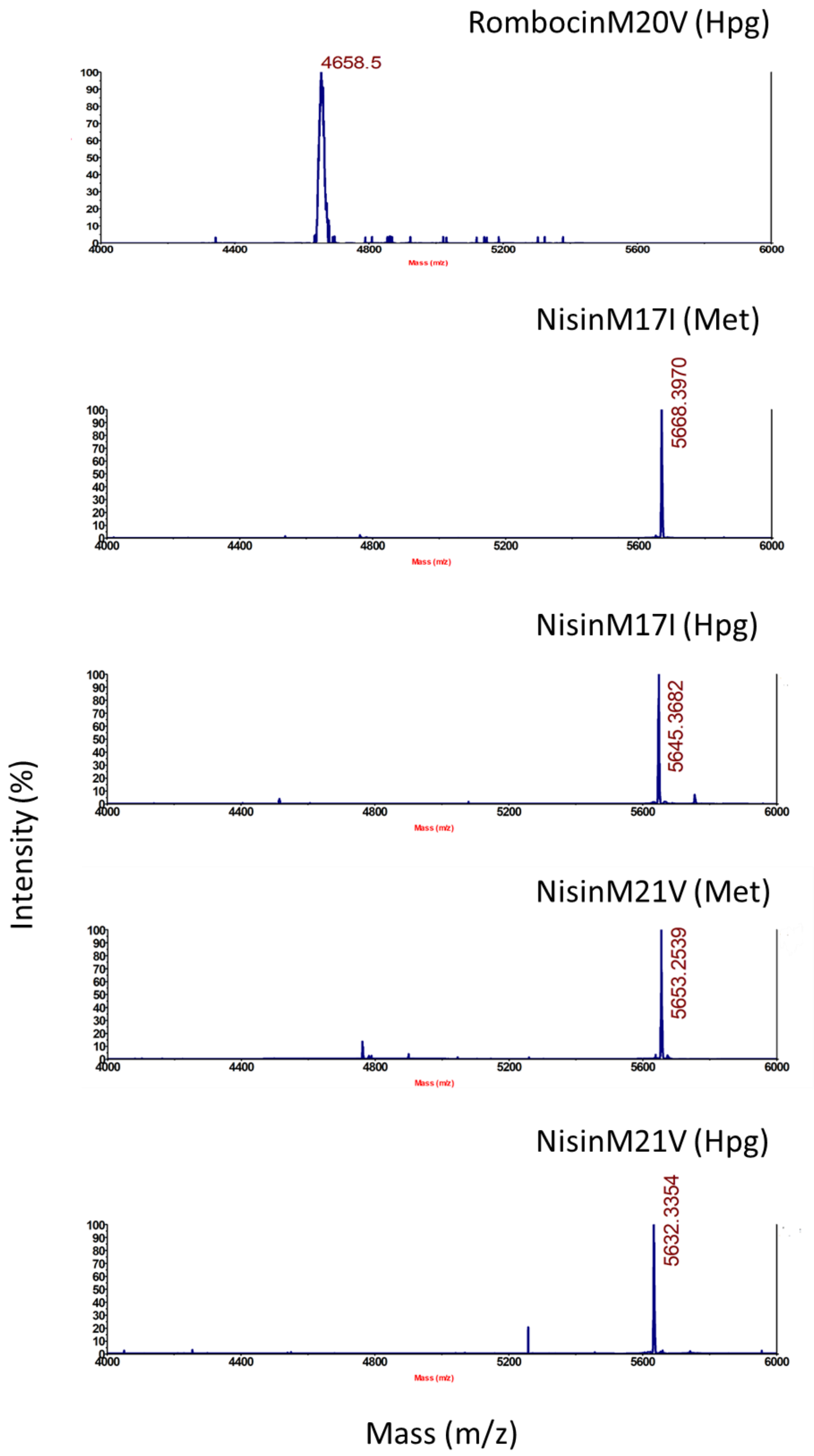

Figure S3 MALDI-TOF MS analysis of nisin variants, obtained from supernatant through TCA precipitation (Figure S2), labeled with Met or the Met analog Homopropargylglycine (Hpg). As presented in Figure S2, not all nisin variants are completely purified after TCA precipitation and this is reflected in some extra MALDI-TOF MS peaks.

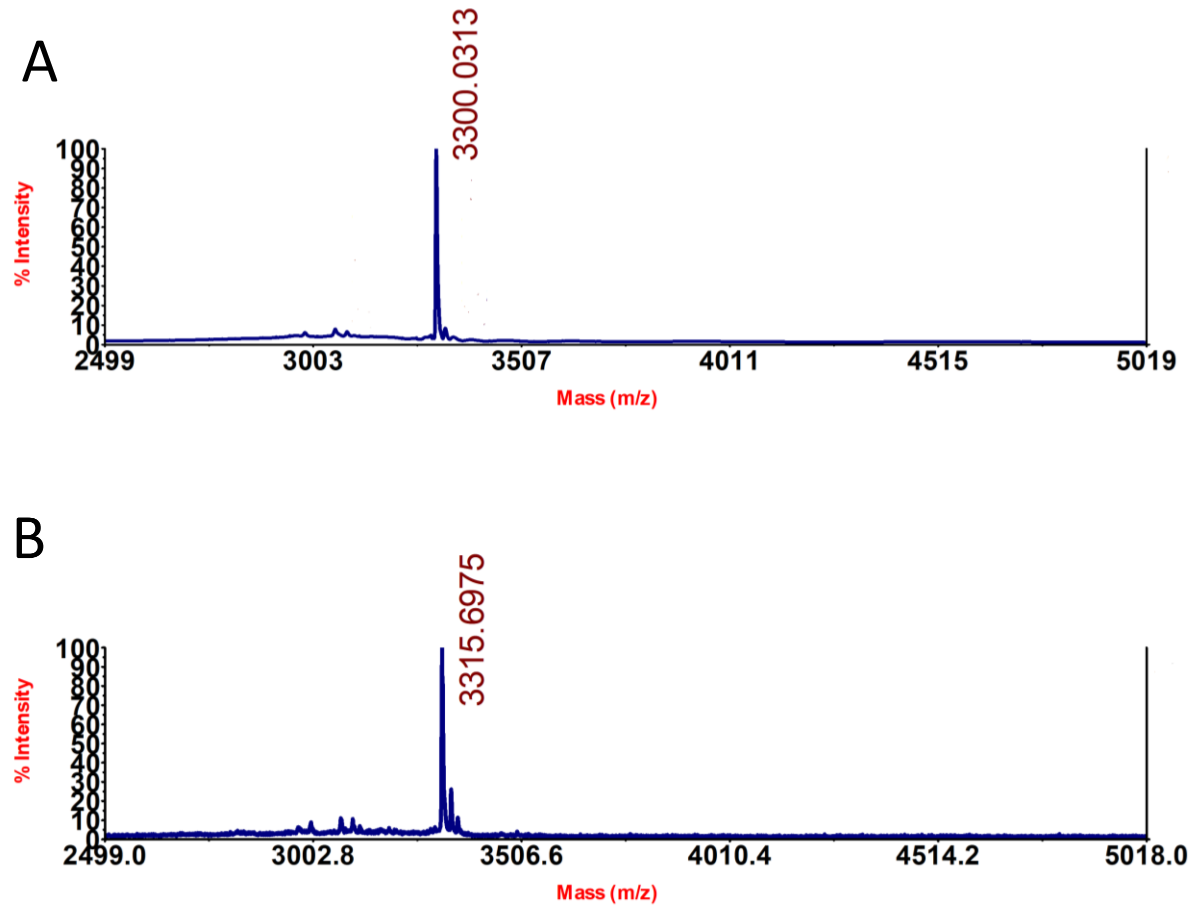

Figure S4 MALDI-TOF mass spectrometry analysis of HPLC-purified nisin variants labeled with Hpg. (**A**) nisin(M21V) has a calculated mass of 3300.00 Da and an observed mass of 3300.03 Da. (**B**) nisin(M17I) has a calculated mass of 3314.03 Da and observed mass of 3315.70 Da.

A

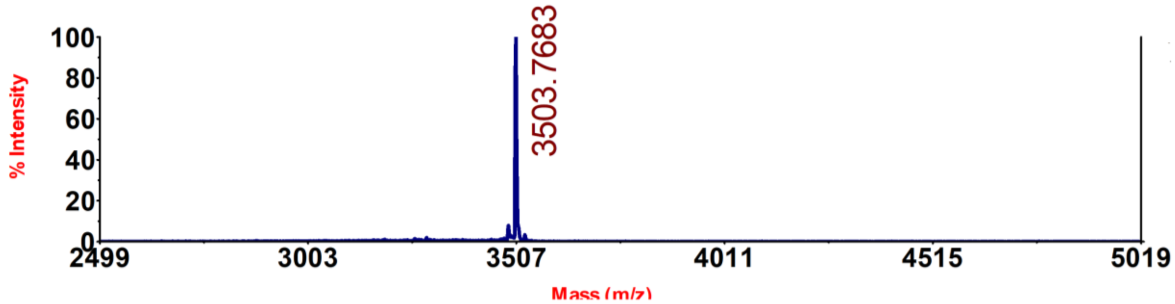

B

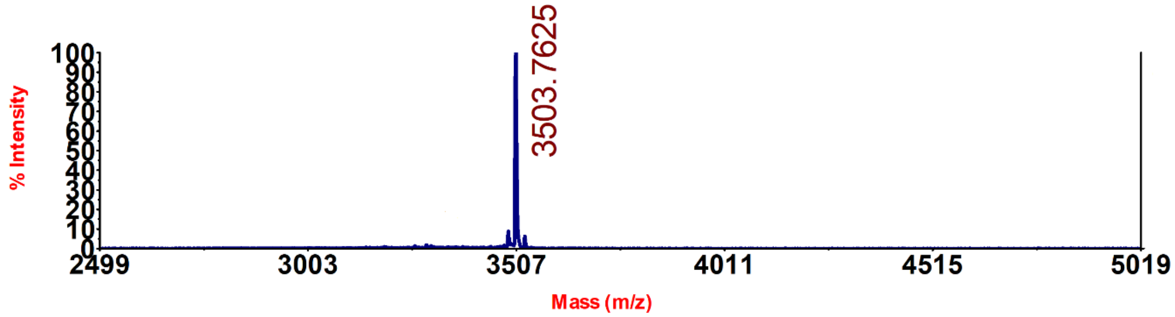

Figure S5 MALDI-TOF mass spectrometry analysis of HPLC-purified nisin(M21V) modified with a Gal or Glc moiety. (**A**) nisin(M21V)-Gal has a calculated mass of 3503.61 Da and observed mass of 3503.77 Da. (**B**) nisinM21V-Glc has a calculated mass of 3503.61 Da and observed mass of 3503.76 Da.

A

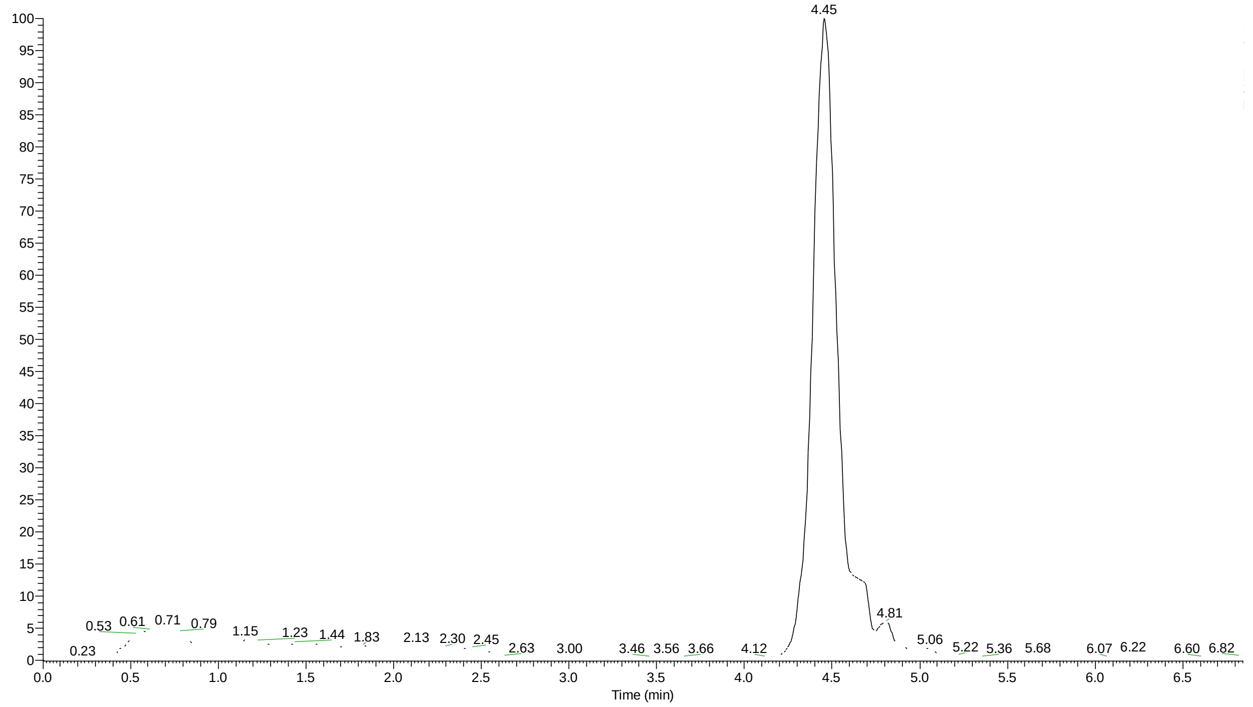
4.45
4.81
Time (min)

B

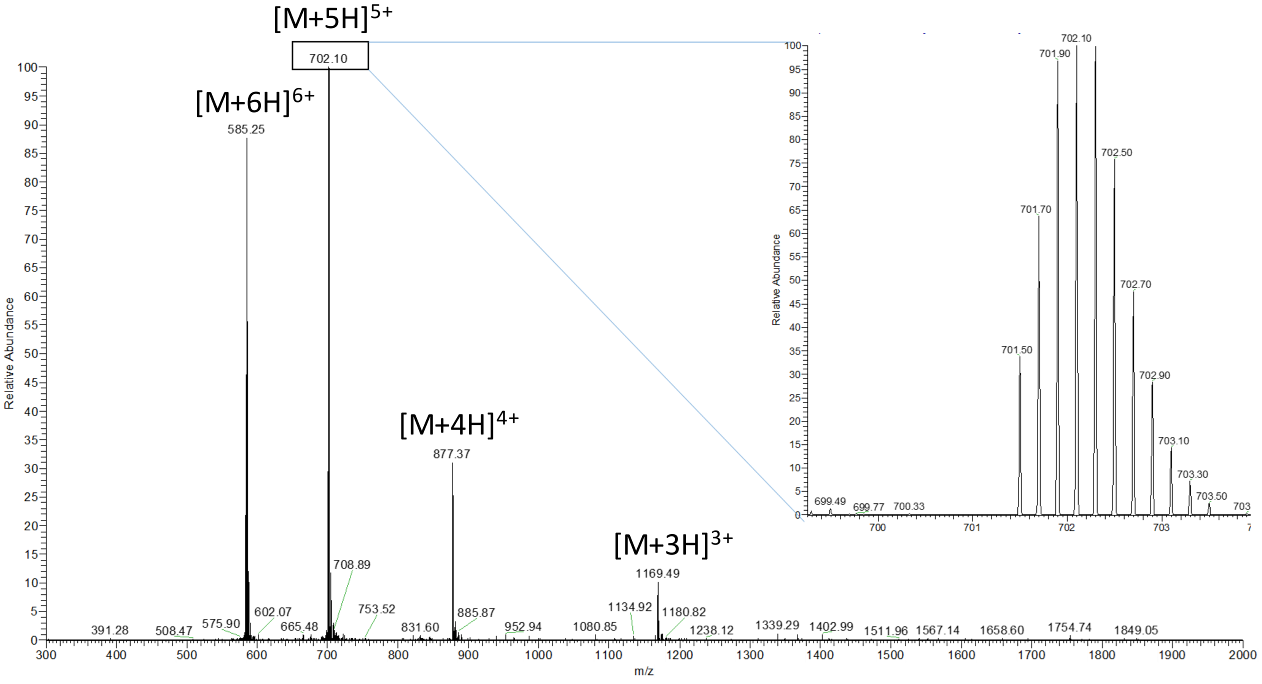
[M+5H]$^{5+}$
702.10
[M+6H]$^{6+}$
585.25
[M+4H]$^{4+}$
877.37
[M+3H]$^{3+}$
1169.49
Relative Abundance
m/z

C

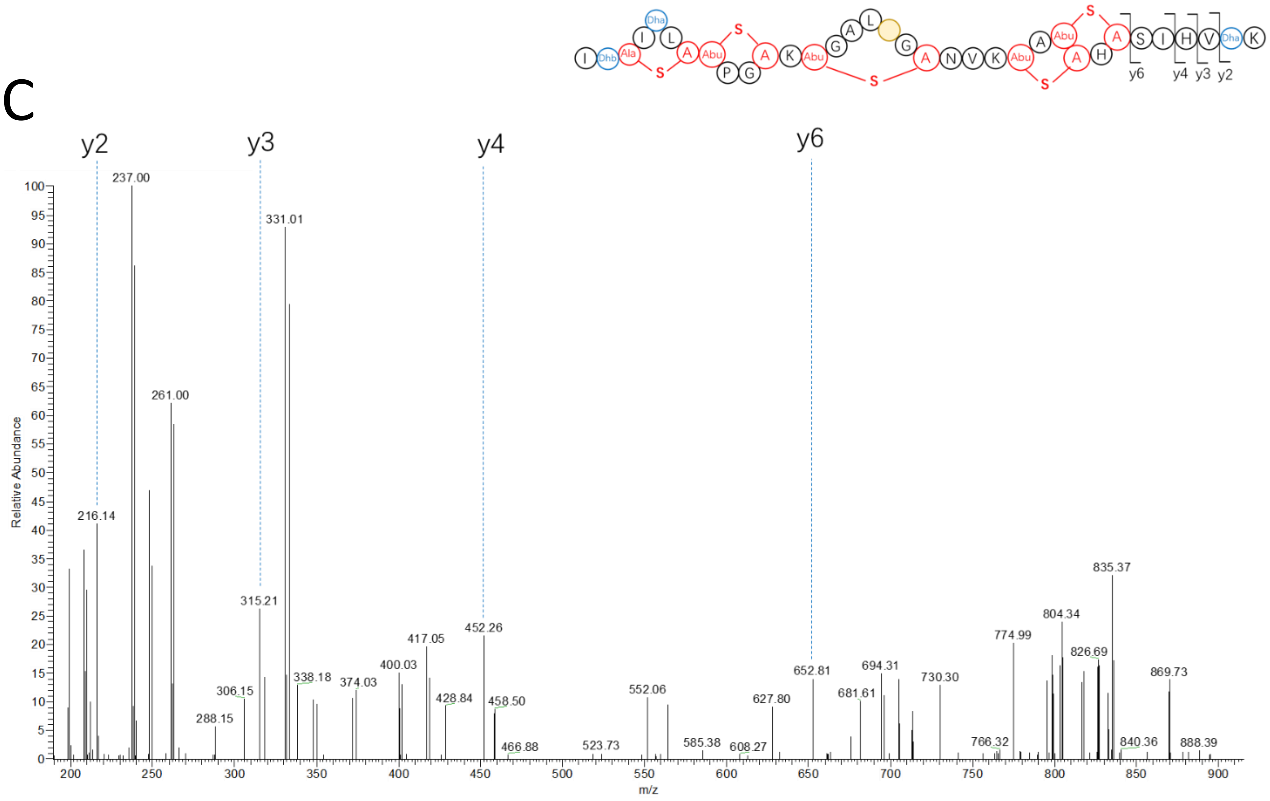

Figure S6 High-Resolution Liquid Chromatography-Mass Spectrometry/Mass Spectrometry (HRLC-MS/MS) Analysis of HPLC-purified nisin(M21V)-Gal. (**A**) HPLC profile of purified nisin(M21V)-Gal. The purity assessment was conducted using a Shimadzu LC20 XR-series HPLC system and an Agilent Persuit XRs C_8 column (50 × 2 mm) at 216 nm. Eluent A (0.1% formic acid in ultra-pure water) and Eluent B (0.1% formic acid in acetonitrile) were used with a flow rate of 0.300 ml/min. The concentration of eluent B varied from 2% to 95% during elution, with the analysis concluding at 10.0 min. (**B**) High-resolution MS spectra of nisin(M21V)-Gal. The calculated mass for $[M+5H]^{5+}$ was 701.53 Da, while the observed mass was 701.50 Da. (**C**) LC-MS/MS spectrum of nisin(M21V)-Gal.

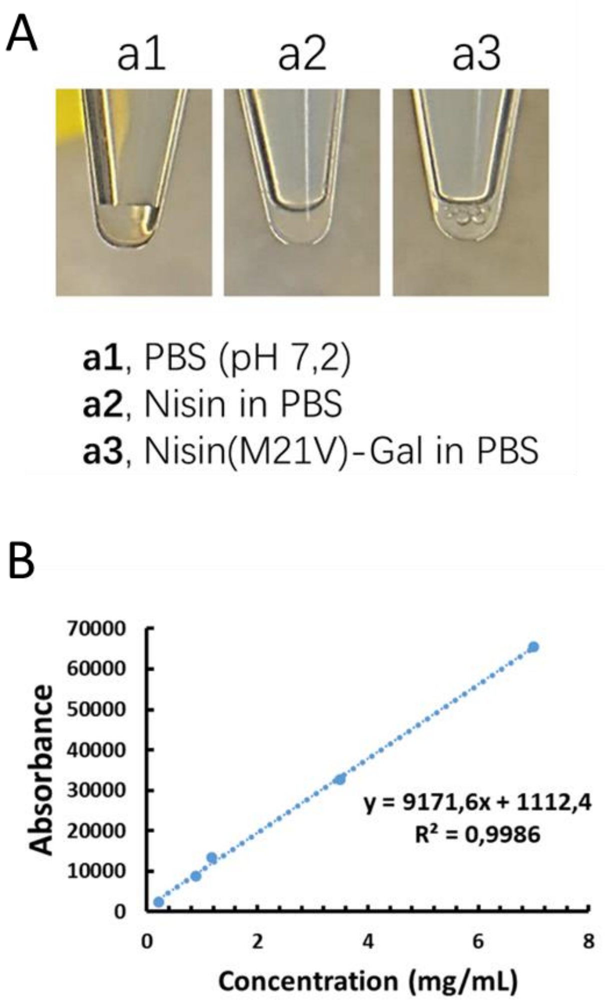

Figure S7 Solubility comparison of nisin and nisin(M21V)-Gal at neutral pH. (A) Saturated nisin and nisin(M21V)-Gal in 50 mM PBS buffer, pH 7.2. (B) HPLC-based determination of a standard nisin concentration curve at 226 nm , with nisin dissolved in a 0.05% acetic acid solution at pH 4.

Table S1 Bacterial strains and plasmids used in this study.

Strains or plasmids	Characteristics	Reference
Strain		
Lactococcus lactis NZ9000	Plasmid construction and maintenance	1
Bacillus cereus CH-85	indicator strain	Lab collection
Listeria monocytogenes LMG10470	indicator strain	Lab collection
Staphylococcus aureus LMG15975	indicator strain, MRSA	Lab collection
Enterococcus faecium LMG16003	indicator strain, VRE	Lab collection
Enterococcus faecalis LMG16216	indicator strain, VRE	Lab collection
plasmid		
pTLReBTC	*nisBTC*, encoding nisin modification machinery, P_{czcD} promoter, Ery^R	2
pNZ-nisA	*nisA*, encoding NisA, P_{nisA} promoter, Cm^R	3
pNZ-rombocin	encoding rombocin A, P_{nisA} promoter, Cm^R	4
pNZ-cesin	encoding cesin A, P_{nisA} promoter, Cm^R	5
pNZnisP8H	*nisP*, encoding NisP mutant, with 8 histidines, Cm^R	6
pNZ-nisin(M17I)	Nisin mutation, P_{nisA} promoter, Cm^R	2
pNZ-nisin(M21V)	Nisin mutation, P_{nisA} promoter, Cm^R	2
pNZ-rombocin(M17I)	Rombocin mutation, P_{nisA} promoter, Cm^R	This study
pNZ-rombocin(M20V)	Rombocin mutation, P_{nisA} promoter, Cm^R	This study

Table S2 Primers used in this study.

Primer	Template	Nucleic acid sequences (5' to 3')	Characteristic
romM17I_fwd	pNZ-rombocin	TACATGTATGAGTAACGGTTGTAAAT	5'- phosphorylation
romM17I_rev		ATAATTACTCCTGTAATACAACCTGC	
romM20V_fwd	pNZ-rombocin	GTAAGTAACGGTTGTAAATAAGCTTTC	5'- phosphorylation
romM20V_rev		ACATGTCATAATTACTCCTGTAATAC	
pNZ-f		TATGAGATAATGCCGACTGTACTTTTTAC	

References

1. Kuipers, O. P., de Ruyter, P. G., Kleerebezem, M., de Vos, W. M. (**1997**). Controlled overproduction of proteins by lactic acid bacteria. *Trends. Biotechnol.*, *15* (4), 135-140.

2. Guo, L., Wang, C., Broos, J., Kuipers, O. P. (**2023**). Lipidated variants of the antimicrobial peptide nisin produced via incorporation of methionine analogs for click chemistry show improved bioactivity. *J. Biol. Chem.*, *299* (7), 104845.

3. van Heel, A. J., Mu, D., Montalbán-López, M., Hendriks, D., Kuipers, O. P. (**2013**). Designing and producing modified, new-to-nature peptides with antimicrobial activity by use of a combination of various lantibiotic modification enzymes. *ACS Synth. Biol.*, *2* (7), 397-404.

4. Guo, L., Wambui, J., Wang, C., Broos, J., Stephan, R., Kuipers, O. P. (**2024**). Rombocin, a short stable natural nisin variant, displays selective antimicrobial activity against Listeria monocytogenes and employs a dual mode of action to kill target bacterial strains. *ACS Synth. Biol.*, *13* (1), 370-383.

5. Guo, L., Wambui, J., Wang, C., Muchaamba, F., Fernandez-Cantos, M. V., Broos, J., Tasara, T., Kuipers, O. P., Stephan, R. (**2023**). Cesin, a short natural variant of nisin, displays potent antimicrobial activity against major pathogens despite lacking two C-terminal macrocycles. *Microbiol. Spectr.*, *11* (5), e05319-22.

6. Montalbán-López, M., Deng, J., Van Heel, A. J., Kuipers, O. P. (**2018**). Specificity and application of the lantibiotic protease NisP. *Front. Microbiol.*, *9*, 160.
